# Structural Elucidation of Three Novel Kaempferol *O-tri*-Glycosides that Are Involved in the Defense Response of Hybrid *Ornithogalum* to *Pectobacterium carotovorum*

**DOI:** 10.3390/molecules24162910

**Published:** 2019-08-10

**Authors:** Iris Yedidia, Keren Schultz, Avner Golan, Hugo E. Gottlieb, Zohar Kerem

**Affiliations:** 1 Department of Ornamental Horticulture, ARO, Volcani Center, Derech Hamacabim 20 P.O. Box 6, 50250 Bet-Dagan, Israel; 2 Institute of Biochemistry, Food Science and Nutrition, The Robert H Smith Faculty of Agriculture, Food and Environment, The Hebrew University of Jerusalem, P.O. Box 12, 7610001 Rehovot, Israel; 3 Department of Chemistry, Bar Ilan University, 52900 Ramat-Gan, Israel

**Keywords:** flavonoid, kaempferol *O-tri*-glycoside, *Ornithogalum*, *Pectobacterium carotovorum*, soft rot

## Abstract

*Ornithogalum* is an ornamental flowering species that grows from a bulb and is highly susceptible to soft-rot disease caused by *Pectobacterium carotovorum* (Pc). Interspecific hybridization between *O. thyrsoides* and *O. dubium* yielded hybrids with enhanced resistance to that pathogen. The hybrids displayed distinct phenolic-compound profiles with several peaks that were specifically heightened following Pc infection. Three of these compounds were isolated and identified as novel kaempferol *O-tri*-glycosides. The structures of these compounds were elucidated using reversed phase high-performance liquid chromatography (RP-LC), RP-LC coupled to high-resolution mass spectrometry (RP-LC-MS), and nuclear magnetic resonance (NMR) (1D ^1^H and ^13^C, DEPT, HMQC, HMBC, COSY, and NOE), in order to achieve pure and defined compounds data. The new compounds were finally identified as kaempferol 3-*O*-[4-*O*-α-l-(3-*O*-acetic)-rhamnopyranosyl-6-*O*-β-d-xylopyranosyl]-β-d-glucopyranoside, kaempferol 3-*O*-[4-*O*-α-l-(2-*O*-acetic)-rhamnopyranosyl-6-*O*-β-d-xylopyranosyl]-β-d-glucopyranoside and kaempferol 3-*O*-[4-*O*-α-l-(2,3-*O*-diacetic)-rhamnopyranosyl-6-*O*-β-d-xylopyranosyl]-β-d-glucopyranoside.

## 1. Introduction

The genus *Ornithogalum,* also known as the “Star of Beth-Lehem”, belongs to the family *Asparagaceae*, which includes about 250–300 species [1]. These include wild and cultivated species that are widely distributed across Europe, Asia (reaching as far east as Afghanistan), Africa and Madagascar [2,3,4]. Most members of this genus are herbaceous perennials, spring- and summer-flowering bulb plants. In recent decades, the African varieties of the plant (along with some others) have been grown commercially and sold as cut flowers and potted plants in South Africa, the USA, the Netherlands and Israel. The plant’s potential as a cut flower and garden plant is severely hampered by its susceptibility to bacterial soft rot caused by *Pectobacterium carotovorum* (Pc) species. Several attempts have been made to minimize soft-rot disease through the use of plant activators that induce systemic resistance and there have also been efforts to develop resistant clones through molecular and classical breeding [5,6,7]. Such strategies have involved the induction and accumulation of secondary metabolites, resulting in reduced bacterial pressure and multiplication, or direct interference with bacterial virulence [8]. In this context, external application of methyl jasmonate (MJ) has been shown to activate the jasmonic-acid signaling pathway, which plays a central role in the regulation of secondary-metabolite biosynthesis in tomato (*Solanum lycopersicum*) [9,10,11,12]. In *O. dubium* and *Zantedeschia aethiopica* (another ornamental monocot), defense elicitation with exogenous MJ has been shown to reduce disease symptoms and lead to increased accumulation of polyphenolic compounds following Pc infection [6,13].

Plant phenolics are considered to be the most abundant secondary metabolites isolated from plants. To date, over 8000 phenolic structures (with simple molecular structures or polymeric structures) have been discovered [14,15]. Most polyphenols appear in nature as glycosides with one or more glycosidic moieties. They are involved in essential processes, such as growth and reproduction, and many help protect plants from biotic and abiotic stress [14,15,16,17]. Flavonoids are known to play important roles in plant tissues, including providing protection against UV-B radiation, as antioxidants, as antifeedants and as phytoalexins [18,19,20,21,22]. Some flavonoids are synthesized in response to plant pathogens [15,16,21,23]. 

Here, two *Ornithogalum* species, *O. dubium*, which is highly susceptible to soft rot, and *O. thyrosoides*, which is relatively resistant, were crossed to yield interspecific F1 hybrids with different levels of resistance to the soft-rot pathogen. In correlation with their observed resistance to soft rot, these hybrids produced metabolites in response to infection with Pc, and those metabolites exhibited patterns of UV absorption that are typical of flavonoids. Three flavonoids were further purified from leaf extracts and mass spectrometry (MS) and nuclear magnetic resonance (NMR) spectroscopy were used to identify them as three novel kaempferol *O-tri*-glycosides.

## 2. Results and Discussion

**Figure 1:**
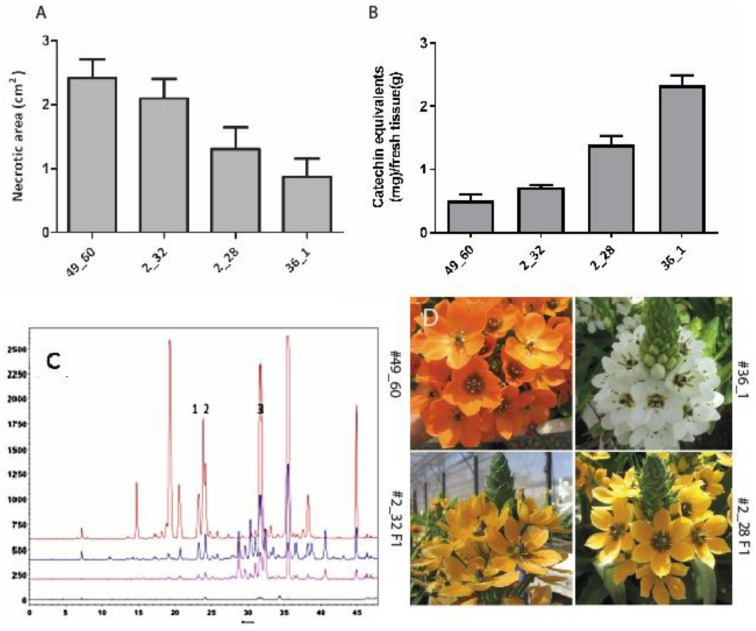
Characterization of *Ornithogalum* breeding lines: Disease development in *Ornithogalum* leaf discs (lines: #49_60, #36_1, #2_28 and #2_32) at 24 h after inoculation with 10 µL of *Pectobacterium carotovorum* suspension (10^8^ CFU/mL). (**A**) Disease severity is expressed as total necrotic area. (**B**) Levels of polyphenolic compounds in methanolic extracts of *Ornithogalum* lines, expressed as catechin equivalents (mg/g fresh tissue). (**C**) RP-LC-UV chromatograms, at 336 nm, of extracts from all *Ornithogalum* lines: Clones #49_60 (black), #2_32 (pink), #2_28 (blue) and #36_1 (brown). (**D**) Flowering of the parental lines and F1 hybrids of the *Ornithogalum* that were used in the study.

Interspecific breeding was carried out between two *Ornithogalum* accessions: *O. dubium* (#49_60) and *O. thyrosoides* (#36_1) [24]. Following embryo rescue, the cross yielded two F1 hybrids, designated #2_28 and #2_32, and micropropagation protocols were used to clone those hybrids (Figure 1D) for further analysis [25]. Infiltration with Pc showed that both hybrids are less sensitive to soft-rot infection than the parent #49_60, with #2_28 being the more resistant (Figure 1A). Following infiltration with Pc, leaves were extracted with aqueous methanol, as described previously [13], and the levels of phenolic compounds in the extracts were determined, revealing an inverse correlation between sensitivity to Pc and levels of phenolics (expressed as catechin equivalents, Figure 1B). All extracts were then separated and characterized using reversed phase high performance liquid chromatography (RP-LC) and photo diode array detection, and a unique profile of phytochemicals was observed for each plant line (Figure 1C). Each of the plant lines had a typical color phenotype: The pollen donor was white, the female flower orange and the F1 hybrids were light orange (Figure 1D). Pc infection was found to induce the production of several compounds as a part of the general plant response to the bacterium with more than two-fold increases in the compounds assigned Peak Numbers 1, 2 and 3. Hyphenated and complementary spectral analyses were used to further characterize these three molecules.

### 2.1. Mass Spectrometry of Compound **1**

**Figure 2:**
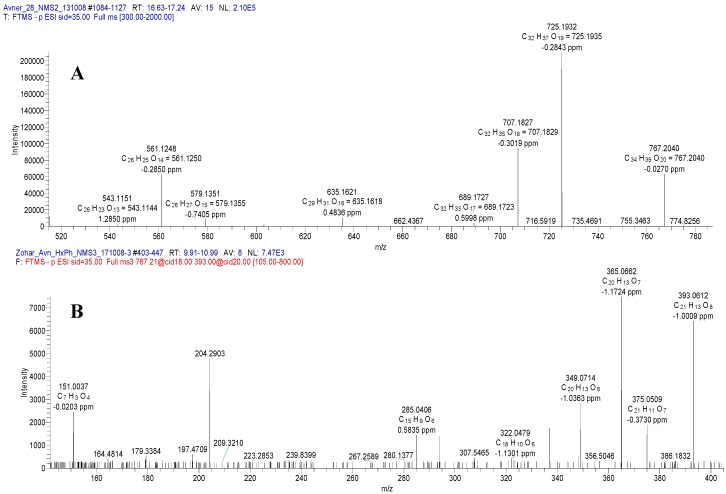
Mass spectra of Compound **1** in the negative mode, full mass spectrum (**A**), and MS^3^ (**B**).

Using ESI-MS (electrospray ionization mass spectrometry) in the negative mode for Compound **1** revealed a molecular ion [M − H]^−^ at *m/z* 767, indicating a molecular formula of C_34_H_39_O_20_, which was confirmed by the observation of the positive mode [M + Na]^+^ ion at *m/z* 791 (Figure 2A). Significant fragment ion peaks, identified by MS^2^ revealed the presence of an ion at *m/z* 725 [767 (M − H) − 42 (CH_3_CO)]^−^, due to a loss of an acetyl moiety, and at 707 [767 (M − H) − 60 (CH_3_CO and water)]^−^, due to additional water removal.

Extensive MS^2^ and MS^3^ fragmentations (Figure 2B), showed the relevant peaks of other important fragments: *m/z* 561 [767 (M − H) − 206 (− acetyl rhamnose (Rha) − H_2_O)]^−^, *m/z* 393 [767 (M − H) − 374 (− acetyl Rha, xylose (Xyl) and 3H_2_O)]^−^ and *m/z* 285 (*m/z* kaempferol aglycone)]. 

### 2.2. NMR Analyses of Compound **1**

**Figure 3:**
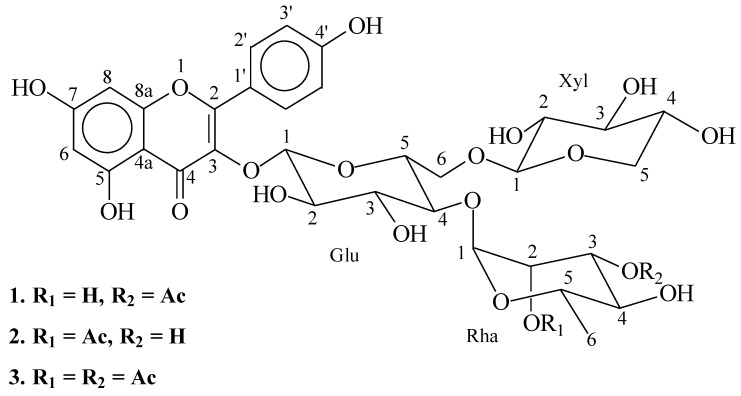
Kaempferol-*O-tri-*glycosides **1**, **2** and **3**.

Additional cleanup of the compound was performed using solid-phase extraction (SPE) through a single-use sep-pak™ C-18, prior to NMR analyses. Indeed, the NMR spectra supported the identification of Compound **1** as kaempferol-*O-tri-*glycoside (**1**, Figure 3): The ^1^H-NMR spectrum of Compound **1** showed four types of aromatic protons [δ 8.08 (2H, *AA*′*XX*′ system), 6.90 (2H, *AA*′*XX*′ system), 6.39 (1H, *d*, *J* = 2 Hz) and 6.18 (1H, *d*, *J* = 2 Hz)] representing the kaempferol aglycone, together with three anomeric protons [δ 5.65 (1H, *d*, *J* = 7.5 Hz, H_Glc_-1), 5.20 (1H, *d*, *J* = 2 Hz, H_Rha_-1) and 4.07 (1H, *d*, *J* = 7.5 Hz, H_Xyl_-1)]. The COSY analysis and anomeric coupling constant values (*J*_Glc,Xyl_ = 7.5 Hz, *J*_Rha_ = 2 Hz) allowed complete identification of the spin systems of β-d glucose (Glc), β-d Xyl and α-l Rha. In addition, 34 carbon signals were observed in the ^13^C-NMR spectrum (Table 1 and Table 2 and Figure 3). Among them, 15 carbons were assigned to the kaempferol unit and three anomeric carbon signals [δ 105.14, 102.70 and 100.26] to the sugar moiety. For the glucose moiety, C_Glc_-4 and C_Glc_-6 were deshielded (δ 71.83, 69.46) due to glycosylation at these two positions. An acetate moiety was also observed.

**Figure 4:**
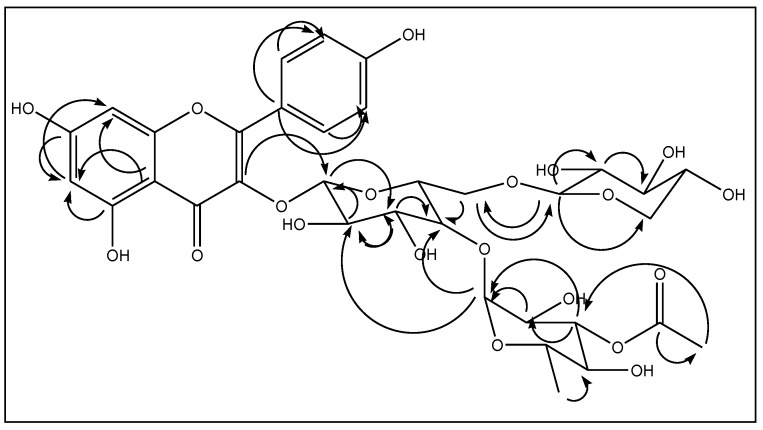
Selected HMBC correlations for compound **1**.

The connectivity of the kaempferol, the three sugar moieties, and the acetate unit was deduced using HMBC correlations (Figure 4). Glc in this molecule was linked to the hydroxyl at C-3 of kaempferol with a ^3^*J*_C-H_ correlation between the H_Glc_-1 and C-3 of the aglycone. It was also deduced from an HMBC cross-peak, that the anomeric protons of Xyl and Rha are correlated with C_Glc_-6 and C_Glc_-4, respectively. Accounting for all the spectroscopic data described above, the structure of **1** was confirmed as kaempferol 3-*O*-[4-*O*-α-l-(3-*O*-acetic)-rhamnopyranosyl-6-*O*-β-d-xylopyranosyl]-β-d-glucopyranoside. 

### 2.3. Spectral Analysis of Compound **2**

Comparison of the mass spectra (ESI-MS) of Compounds **2** and **1** revealed the presence of similar molecular ions in both the positive and the negative modes ([M + Na]^+^ at *m/z* 791 and [M − H]^−^ at *m/z* 767), resulting in the molecular formula C_34_H_39_O_20_. The MS^2^ fragmentation of those molecular peaks was similar, indicating that Flavonoids **1** and **2** are isomers. Data acquired using NMR analyses similar to those described above allowed the identification of Compound **2** as a kaempferol aglycone bound to three monosaccharide units. The ^1^H-NMR spectrum showed four types of aromatic protons [δ 6.89 (2H, *AA’XX’* system), 8.07 (2H, *AA’XX’* system), 6.39 (1H, *d*, *J* = 2 Hz), and 6.17 (1H, *d*, *J* = 2 Hz)] due to kaempferol, along with three anomeric protons [δ 5.64 (1H, *d*, *J* = 7.5 Hz, H_Glc_-1)], 5.21 (1H, *d*, *J* = 2 Hz, H_Rha_-1) and 4.06 (1H, *d*, *J* = 7.5 Hz, H_Xyl_-1)] (Table 1 and Table 2, Figure 3). 

The ^1^H-NMR spectra of Compounds **1** and **2** were almost identical except for signals arising from the rhamnose acetate moiety. In the NMR analysis, it was concluded that Compound **2** is an isomer of Compound **1** in which the acetate group is attached to position 2 rather than position 3 of the rhamnose moiety. To conclude, compound **2** was identified as kaempferol 3-*O*-[4-*O*-α-L-(2-*O*-acetic)-rhamnopyranosyl-6-*O*-β-d-xylopyranosyl]-β-d-glucopyranoside.

### 2.4. Spectral Analysis of Compound **3**

The spectral data acquired for Compound 3 resembled the spectral data for Compounds **1** and **2**. Compound **3** had a molecular ion peak [M − H]^−^ at *m/z* 809 in the negative ESI mode, which indicates a molecular formula of C_36_H_41_O_21_. As above, this was confirmed by the ion [M + H]^+^ at *m/z* 811 in the positive mode. Fragment ion peaks of MS^2^ and MS^3^ at *m/z*: 767 [809 (M − H)-42 (CH_3_CO)]^−^, 561 [809 (M − H) − 248 (-diacetylated rhamnose and H_2_O)]^−^, 393 [809 (M − H) − 416 (-diacetylated rhamnose, xylose and 3H_2_O)]^−^ and 285 [809 (M − H) − 524 (kaempferol aglycone)]. Compound **3** was shown to have a kaempferol aglycone with four aromatic protons [δ 6.91 (2H, *AA’XX’* system), 8.07 (2H, *AA’XX’* system), 6.40 (1H, *d*, *J* = 2 Hz), and 6.19 (1H, *d*, *J* = 2 Hz)], and three anomeric protons [δ 5.64 (1H, *d*, *J* = 7.5 Hz, H_Glc_-1)], 5.20 (1H, *d*, *J* = 2 Hz, H_Rha_-1) and 4.07 (1H, *d*, *J* = 7.5 Hz, H_Xyl_-1)] and three anomeric carbons [100.25, 105.15, 100.07]. Two acetylated positions on Rha were deuced from the two ^1^H-NMR singlets at δ 2.01 and 2.09 (C_Rha_-2 and C_Rha_-3), and confirmed by ^13^C-NMR signals at δ 20.94 and 20.75 (methyls) and δ 171.79 and 172.41 (carbonyls; C_Rha_-2 and C_Rha_-3, respectively; Table 1 and Table 2). These NMR spectra, supported by the results of COSY, DEPT, HMQC, HMBC, and NOE experiments, allowed us to identify Compound **3** as kaempferol 3-*O*-[4-*O*-α-l-(2,3-*O*-diacetic)–rhamnopyranosyl-6-*O*-β−xylopyranosyl]-β-d-glucopyranoside.

## 3. Experimental

### 3.1. General

All solutions were prepared in DDW (double distilled water), unless indicated otherwise. All materials were purchased, from Sigma-Aldrich, unless otherwise indicated. HPLC grade methanol, ethanol, acetonitrile, ethyl acetate, hexane were purchased from Baker (Phillipsburg, NG, USA). All tissue culture materials were purchased from Duchefa (Haarlem, The Netherlands). 

RP-LC-MS analyses were performed using Accela High-Speed LC system coupled with linear trap quadrupole (LTQ) Orbitrap Discovery hybrid mass spectrometer (Thermo Fisher Scientific Inc., Waltham, MA, USA) equipped with electrospray ionization source. The mass spectrometer was operated in both negative and positive ionization modes, and ion source was set as follows: Spray voltage 3 kV, capillary temperature 250 °C, ion-transfer optics parameters were optimized using automatic tune option, sheath gas rate (arb) 35, and auxiliary gas rate (arb) 15. Mass spectra were acquired in the *m/z* 150–2000 Da range. The LC-MS^3^ analysis was performed in data depending acquisition mode. Data were analyzed using Xcalibur software (Thermo Fisher Scientific Inc., Waltham, MA, USA, version 1.4 SR1).

All NMR spectra (1D ^1^H and ^13^C, COSY, DEPT, HMQC, HMBC and NOE), were recorded on a Bruker Avance-III-700 spectrometer (Bruker, Germany). Chemical shifts are reported in δ units with TMS (tetramethylsilane) as the internal standard.

### 3.2. Plant Material, Establishment of Cell Cultures, Plants and Bacterial Infection

Two species of *Ornithogalum*, *O. dubium* (#49_60) and *O. thyrsoides* (#95/36/1) were crossed to produce F1 interspecific hybrids, of which two lines were selected designated as #2_28 and #2_32 and cloned for further analyses [24]. Cell cultures and infection methods were executed according to previous works [6,13]. Briefly, 20 micropropagated plantlets of each of the F1 hybrids were inoculated by spraying 1 mL of fresh cultures of Pc 10^6^ CFU mL^−1^ in a laminar flow hood. Bacterial cell cultures were washed twice and re-suspended in DDW before inoculation. 

### 3.3. Extraction and Separation 

Leaves of *Ornithogalum* were obtained from either greenhouse mature plants or plantlets from tissue cultures. The extraction of phenolics was executed 48 h post-inoculation of plantlets, and samples (20 g) were frozen in liquid nitrogen, ground and dried. Selective extraction of phenolic compounds from plant tissues was adjusted based on previous works [13,26,27]. Dry samples were suspended in an acidic methanol solution (100 mL/sample, MeOH_80%/_HCl_0.1%)_, and kept stirred at 4 °C overnight. The supernatant was filtered through a double layer glass fiber (Whatman, 47 mm, 0.2 μm and Whatman GF/C 47 mm), acidified (pH = 2), and washed with hexane (x3, *v/v*). The aqueous layer was washed again with ethyl acetate (x3, *Vol/Vol*), to remove free phenolics (aglycones). The aqueous phase was evaporated to dryness under vacuum and reconstituted using 10 mL of methanol (HPLC grade), to a final concentration of 2 mg/mL. The samples were kept frozen (−20 °C) for future analyses. 

### 3.4. Reversed Phase High-Performance Liquid Chromatography (RP-LC) Assay of Kaempferol 

Fractions, isolated and collected by flash chromatography, were analyzed using an RP-LC system: TSP P4000 (Thermo Separation Products, Riviera Beach, FL, USA), consisting of an auto sampler (AS3000), pump (P3000), injector (100 µL), column oven (30 °C) and diode-array detector (UV6000). Prior to analysis, 100 μL of extracted sample in MeOH, was added to 100 μL of DDW. A reversed-phase (RP) C-18 column (Phenomenex, Luna, C18, 250 × 4.60 mm, 5 μm) was employed. Gradient elution was performed using water (A) and MeOH-ACN (1:1) (B). Initial conditions were 72% A, a linear gradient to 64% A from 2 min to 24 min, a second linear gradient to 100% B for 2 min, and held for 4 min at 100% B, at a flow rate of 1 mL/min. Absorption of flavonoids was monitored at 336 nm.

### 3.5. Solid Phase Extraction (SPE)

Pure compounds for spectral analyses were prepared using 50 mg/0.5 mL sep-pak™ C-18 columns (Strata, Phenomenex, Torrance, CA, USA), following RP-LC separations. Prior to use, columns were pre-conditioned with 5.0 mL of methanol, followed by 5.0 mL of double distilled water and finally 5.0 mL of 10% methanol in water. Before being loaded onto the column, aqueous methanol solution (10% volume) was added to each dried RP-LC fraction. Fractions (100 µL) were then eluted with MeOH (GC grade), UV absorbing fractions were dried under nitrogen stream. 

### 3.6. Kaempferol O-tri-glycoside (**1**)

White amorphous powder: UV λ_max_ (MeOH): 265, 350 nm; ESI-MS (negative mode) *m/z*: 767.2041 [M − H]^−^; ESI-MS (positive mode) *m/z*: 791.1 [M + Na]^+^; ^1^H-NMR (methanol-*d_4_*, 700 MHz) and ^13^C-NMR (methanol-*d_4_*, 176 MHz) spectral data (see Table 1 and Table 2).

### 3.7. Kaempferol O-tri-glycoside (**2**)

White amorphous powder: UV λ_max_ (MeOH): 265, 350 nm; ESI-MS (negative mode) *m/z*: 767.2041 [M − H]^−^; ESI-MS (positive mode) *m/z*: 791.1 [M + Na]^+^; ^1^H-NMR (methanol-*d_4_*, 700 MHz) and ^13^C-NMR (methanol-*d_4_*, 176 MHz) spectral data (see Table 1 and Table 2).

### 3.8. Kaempferol O-tri-glycoside (**3**)

Yellow amorphous powder: UV λ_max_ (MeOH): 265, 350 nm; ESI-MS (negative mode) *m/z*: 809.2145 [M − H]^−^; ESI-MS (positive mode) *m/z*: 811.2279 [M + H]^+^; ^1^H-NMR (methanol-*d_4_*, 700 MHz) and ^13^C-NMR (methanol-*d_4_*, 176 MHz) spectral data (see Table 1 and Table 2).

## 4. Conclusions

The induction of the synthesis of flavonoids in *Ornithogalum* hybrids following infection with the soft-rot pathogen Pc revealed three novel kaempferol *O-tri*-glycosides. The levels of these compounds correlated with increased resistance to Pc infection in the parent line *O. thyrosoides* (#36_1), and in the F1 hybrids #2_28 and #2_32. The results suggest that interspecific breeding may be a practical approach to fight bacterial soft rot in ornamental flower bulbs.

## Figures and Tables

**Table 1:** ^13^C- and ^1^H-NMR data (*J* in Hz) for the aglycone moiety of kaempferol glycosides **1**, **2** and **3** in CD_3_OD.

	1		2		3	
	^13^C	^1^H	^13^C	^1^H	^13^C	^1^H
**Kaempferol**						
**2**	158.54	—	158.57	—	158.61	—
**3**	134.53	—	134.51	—	134.56	—
**4**	179.38	—	179.32	—	179.37	—
**4a**	106.04	—	106.00	—	106.13	—
**5**	163.23	—	163.22	—	163.25	—
**6**	99.84	6.18 (*d*, 2)	99.89	6.17 (*d*, 2)	99.80	6.19 (*d*, 2)
**7**	165.90	—	165.83	—	165.69	—
**8**	94.80	6.39 (*d*, 2)	94.87	6.39 (*d*, 2)	94.79	6.40 (*d*, 2)
**8a**	158.49	—	158.47	—	158.54	—
**1′**	123.10	—	123.08	—	123.09	—
**2′, 6′**	132.24	8.08 (*AA’XX’*)	132.24	8.07 (*AA’XX’*)	132.23	8.07 (*AA’XX’*)
**3′, 5′**	116.20	6.90 (*AA’XX*’)	116.16	6.89 (*AA’XX’*)	116.22	6.91 (*AA’XX’*)
**4′**	161.41	—	161.42	—	161.43	—

**Table 2:** ^13^C and ^1^H-NMR data (*J* in Hz) for the sugar units of kaempferol glycosides **1**, **2** and **3** in CD_3_OD.

	1		2		3	
	^13^C	^1^H	^13^C	^1^H	^13^C	^1^H
**Glucose**						
**1**	100.26	5.65 (*d*, 7.5)	100.34	5.64 (*d*, 7.5)	100.25	5.64 (*d*, 7.5)
**2**	80.51	3.61 (*dd*, 9.5,7.5)	79.72	3.60 (*dd*, 9.5,7.5)	80.41	3.62 (*dd*, 9.5,7.5)
**3**	78.60	3.57 (*dd*, 9.5, 9)	78.57	3.57 (*dd*, 9.5, 9)	78.44	3.57 (*dd*, 9.5, 9)
**4**	71.83	3.31 ^a^	71.84	3.29 ^a^	71.85	3.30 ^a^
**5**	77.58	3.41 (*ddd*, 9.5, 6, 2)	77.58	3.40 (*ddd*, 9.5, 6, 2)	77.58	3.41 (*ddd*, 9.5, 6, 2)
**6**	69.46	3.57 (*dd*, 12, 6)	69.47	3.56 (*dd*, 12, 6)	69.47	3.58 (*dd*, 12, 6)
		3.90 (*dd*, 12, 2)		3.90 (*dd*, 12, 2)		3.90 (*dd*, 12, 2)
**Xylose**						
**1**	105.14	4.07 (*d*, 7.5)	105.14	4.06 (*d*, 7.5)	105.15	4.07 (*d*, 7.5)
**2**	74.76	2.99 (*dd*, 9, 7.5)	74.75	2.99 (*dd*, 9, 7.5)	74.75	3.00 (*dd*, 9, 7.5)
**3**	77.42	3.08 (*t*, 9)	77.42	3.09 (*t*, 9)	77.41	3.09 (*t*, 9)
**4**	71.02	3.34 ^a^	71.02	3.33 ^a^	71.04	3.34 ^a^
**5**	66.54	2.88 (*dd*, 11.5, 10)	66.54	2.87 (*dd*, 11.5, 10)	66.53	2.88 (*dd*, 11.5, 10)
		3.64 (*dd*, 11.5, 5)		3.64 (*dd*, 11.5, 5)		3.65 (*dd*, 11.5, 5)
**Rhamnose**						
**1**	102.70	5.20 (*d*, 2)	100.25	5.21 (*d*, 2)	100.07	5.20 (*d*, 2)
**2**	69.99	4.18 (*dd*, 3.5, 2)	74.22	5.23 (*dd*, 3.5, 2)	71.44 ^b^	5.42 (*dd*, 3.5, 2)
**3**	75.98	5.05 (*dd*, 10, 3.5)	70.52	3.97 (*dd*, 10, 3.5)	73.50	5.18 (*dd*, 10, 3.5)
**4**	71.30	3.55 (*t*, 10)	74.27	3.30 (*t*, 10)	71.51 ^b^	3.46 (*t*, 10)
**5**	70.10	4.20 (*dd*, 10, 6)	70.10	4.10 (*dd*, 10, 6)	70.02	4.24 (*dd*, 10, 6)
**6**	17.58	1.00 (*d*, 6, 3H)	17.61	1.00 (*d*, 6, 3H)	17.55	1.02 (*d*, 6, 3H)
**2-Ac**	—	—	20.98	2.09 (*s*, 3H)	20.94	2.01 (*s*, 3H)
	—	—	172.31	—	171.79	—
**3-Ac**	21.16	2.11 (*s*, 3H)	—	—	20.75	2.09 (*s*, 3H)
	172.82	—	—	—	172.41	—

^a^ Signal under solvent residual peak. ^b^ Chemical shifts may be interchanged.
